# Associations of seminal plasma metal mixtures with sperm quality parameters and exploratory mediation analysis of urinary oxidative stress biomarkers: a cross-sectional study from the RHCC-AC preconception cohort

**DOI:** 10.3389/fendo.2026.1861843

**Published:** 2026-07-08

**Authors:** Xuemei Wang, Caiyun Wu, Lin Su, Jing Wang, Yu Li, Feng Ni, Hong Jiang

**Affiliations:** 1 Reproductive Medicine Center, Clinical College of People’s Liberation Army Affiliated to Anhui Medical University, Hefei, China; 2 Reproductive Medicine Center, The 901th Hospital of the Joint Logistics Support Force of People’s Liberation Army, Hefei, China

**Keywords:** mediation analysis, metal mixtures, oxidative stress, seminal plasma metals, sperm quality

## Abstract

**Background:**

There is increasing concern over the global decline in semen quality. However, the role of environmental metal exposure remains uncertain, and the underlying mechanisms are poorly understood.

**Methods:**

A cross−sectional cohort study included 425 men from the Reproductive Health of Childbearing Couples–Anhui Cohort (RHCC-AC). Seventeen metals in seminal plasma were quantified by ICP−MS. Eight urinary oxidative stress biomarkers were measured by UPLC−MS/MS. Sperm quality parameters included sperm concentration, progressive motility, total motility, abnormality rate, and sperm DNA fragmentation index (DFI). Weighted quantile sum (WQS) regression was applied to assess single and joint effects of metal mixtures. In addition, exploratory mediation analysis was conducted to examine the potential mediating role of oxidative stress in the associations between metal exposure and sperm quality.

**Results:**

Single−metal analyses showed that Al was positively associated with sperm abnormality rate, whereas Co and Cu were inversely associated with progressive and total motility. As, Fe, and Se were positively associated with sperm concentration. In mixture analyses, Al was the primary contributor to increased sperm abnormality rate (positive−direction WQS: *β* = 0.014; 95% *CI*: 0.004–0.024; Al weight = 0.357). Co was the main contributor to reduced total motility (*β* = −0.188; 95% *CI*: −0.309 to −0.067; Co weight = 0.419). Few significant associations were observed between metals and oxidative stress biomarkers. Among oxidative stress markers, dityrosine(diY) was positively associated with sperm abnormality rate, while D,L-ortho-tyrosine(D,L-o-tyrosine) was negatively associated with sperm concentration. Exploratory mediation analysis revealed that D,L-o-tyrosine significantly mediated the associations of seminal plasma Hg and V with sperm concentration, with mediated proportions of 11.8% and 13.0%, respectively.

**Conclusion:**

Seminal plasma metal mixtures, particularly Al and Co, were associated with sperm quality. Exploratory mediation analysis suggested potential indirect effects of D,L-o-tyrosine in the associations of Hg and V with sperm concentration, but these findings should be interpreted cautiously. Further studies using seminal or reproductive tract-specific oxidative stress biomarkers are warranted to clarify the role of oxidative stress in linking metal exposure to male reproductive system injury.

## 1 Introduction

Infertility affects approximately 15% of couples worldwide, and male factors account for 40–50% of infertility cases (1). Sperm quality, including parameters such as sperm concentration, motility, and morphology, is widely recognized as a key clinical indicator of male fertility (2). Epidemiological evidence has documented a substantial long-term decline in sperm concentration, with meta-analytic data indicating an approximately 50–60% reduction between 1973 and 2011 (3). Despite considerable progress in reproductive medicine, the etiology of poor sperm quality remains incompletely understood. Epidemiological studies have identified advanced age, environmental and occupational exposures, dietary factors, and lifestyle behaviors as key risk factors associated with impaired sperm quality (4). Nevertheless, these recognized determinants do not fully account for the observed variability and decline in semen quality (5). This gap highlights the possibility that additional factors or underlying mechanisms may influence sperm quality.

Rapid industrialization and globalization have led to widespread contamination of air, water, and soil. Among the various environmental pollutants identified, metals derived from anthropogenic activities—such as industrial processes, traffic emissions, and energy production—have attracted increasing attention because of their persistence and potential adverse effects on human health (6) Metals—including essential metals, heavy metals, and metalloids—are commonly co-occurring in the environment and often exhibit correlated exposure patterns (7). Unlike essential trace elements, most heavy metals are generally considered to lack physiological functions in humans and may exert toxic effects even at relatively low concentrations (8). Epidemiological and experimental studies have suggested that exposure to heavy metals or metalloids is associated with impaired sperm quality. For example, Chai et al. reported that vanadium (V) and nickel (Ni) were negatively associated with normal sperm morphology (9). Additionally, previous studies by Tvrda et al. and Liu et al. have suggested that excessive exposure to essential metals such as iron (Fe), copper (Cu), and manganese (Mn) may be associated with impaired sperm quality (10, 11).

Despite increasing evidence linking metal or metalloid exposure to semen quality, relatively few studies have directly measured these elements in seminal plasma. Given that seminal plasma constitutes the immediate microenvironment surrounding spermatozoa and that trace element distributions vary substantially across biological fluids, assessing metal exposure using seminal plasma samples may provide more biologically relevant insight into the relationship between metal exposure and sperm quality.

Oxidative stress is defined as a state resulting from an imbalance between the production of reactive oxygen species (ROS) and antioxidant defenses (12). Common biomarkers of oxidative stress include 8-hydroxy-2′-deoxyguanosine (8-OHdG), 8-hydroxyguanosine (8-OHG), 4-hydroxynonenal mercapturic acid (HNEMA), dityrosine (diY), and D,L-ortho-tyrosine(D,L-o-tyrosine). These biomarkers are widely used to assess oxidative damage at the molecular level, reflecting oxidative modifications to DNA, RNA, lipids, and proteins (13–15). Oxidative stress can compromise the structural and functional integrity of sperm and has been widely implicated in sperm dysfunction and male infertility (16). Supporting this, elevated levels of oxidative damage biomarkers, such as 8-OHdG, have been inversely associated with normal sperm morphology and total sperm count (17). Similarly, increased oxidative damage markers in seminal plasma have been positively associated with a higher proportion of morphologically abnormal sperm (18).

Environmental metal exposure can disrupt redox homeostasis, and metals such as arsenic (As) and lead (Pb) have been reported to induce oxidative stress by promoting reactive oxygen species (ROS) generation (19). However, evidence regarding the mediating role of oxidative stress biomarkers in the association between metal exposure and impaired sperm quality remains limited. He et al. (17) reported that 8-OHdG mediated the association between lead exposure and sperm abnormality rate, whereas 8-isoPGF2α mediated the associations of As and cadmium exposure with reduced sperm motility. These findings support oxidative stress as a biologically plausible mechanism linking metal exposure to impaired male reproductive function, but its potential mediating role in the association between seminal metal exposure and sperm quality remains to be clarified.

To address these gaps, we conducted an integrated investigation of the relationship between seminal plasma metal exposure and sperm quality in men of reproductive age. Using data from a subset of the Reproductive Health of Childbearing Couples—Anhui Cohort (RHCC-AC), we applied complementary mixture modeling approaches to evaluate the effects of individual metals and metal mixtures on sperm quality and to identify key contributing metals. We further assessed associations between urinary oxidative stress biomarkers and semen quality parameters and explored whether urinary biomarkers can serve as a biologically relevant matrix for evaluating oxidative stress in relation to seminal plasma metal exposure and sperm quality.

This integrated framework may improve understanding of the biological pathways linking environmental metal exposure to impaired sperm quality and may facilitate the identification of clinically relevant, non-invasive biomarkers for early fertility risk assessment and infertility screening in men of reproductive age.

## 2 Materials and methods

### 2.1 Study population

Participants were drawn from the Reproductive Health of Childbearing Couples–Anhui Cohort (RHCC-AC), a large-scale prospective multicenter preconception cohort investigating environmental and lifestyle determinants of fertility and offspring development. Baseline information on the male partner, including age, educational level, smoking status, alcohol consumption, body mass index (BMI), and infertility type, was collected using standardized questionnaires. We excluded 154 participants without valid urine samples, for whom urinary oxidative stress biomarkers could not be assessed. The final analytical sample consisted of 425 men.

### 2.2 Sample collection and semen analysis

Semen samples were obtained by masturbation in a private room after a self-reported abstinence period of 2–7 days. After collection, some of the samples were immediately liquefied at 37 °C and then analyzed by professional technicians according to the guideline of the WHO laboratory manual for semen examination (WHO 2010b). The other part of the samples was aliquoted into Eppendorf tubes and stored at −80 °C until the analysis of metal concentrations was conducted. Before analysis, samples were thawed overnight at 4 °C and centrifuged at 3000 × g for 15 min to separate seminal plasma. Concentrations of aluminum (Al), vanadium (V), chromium (Cr), manganese (Mn), iron (Fe), cobalt (Co), nickel (Ni), copper (Cu), arsenic (As), selenium (Se), strontium (Sr), cadmium (Cd), tin (Sn), barium (Ba), mercury (Hg), thallium (Tl), and lead (Pb) in seminal plasma were quantified using inductively coupled plasma mass spectrometry (ICP-MS; NexION 350X, PerkinElmer, Shelton, CT, USA). Prior to measurement, samples were thoroughly mixed on a shaker for at least 1 h, followed by acid digestion with 200 μL nitric acid at 105 °C for 2 h. After cooling, the digested samples were diluted 1:50 with 0.05% Triton X-100 before ICP-MS analysis. Instrumental drift was corrected using internal standards, and calibration curves were established with multi-element standard solutions (10 μg/mL; PerkinElmer, USA). The coefficients of determination (R²) for all metals exceeded 0.999. Limits of detection (LODs) is provided in **Supplementary Table 1**.

### 2.3 Urinary oxidative stress assessment

Ultrahigh-performance liquid chromatography–tandem mass spectrometry (UPLC–MS/MS) was used to quantify eight urinary oxidative stress (OS) biomarkers. Quantification was performed using an isotope-labeled internal standard approach. The biomarker panel included 8-hydroxy-2′-deoxyguanosine (8-OHdG) as an indicator of DNA oxidative damage; 8-hydroxyguanosine (8-OHG) and 8-hydroxyguanine (8-OHGua) as markers of RNA oxidative damage; 4-hydroxy-2-nonenal-mercapturic acid (HNE-MA) as a lipid peroxidation marker; dityrosine (diY) and D,L-ortho-tyrosine (D,L-o-tyrosine) as indicators of protein oxidative damage; N^6^-carboxyethyl-lysine (CEL) as an advanced glycation end product; and allantoin (Alla) as a marker of uric acid oxidation. Briefly, 100 μL urine was mixed with β-glucuronidase/arylsulfatase and isotope-labeled internal standards and incubated overnight at 37 °C. After incubation, samples were diluted 1:5 (v/v) with 0.05% formic acid in water, centrifuged at 11,000 × g for 30 min, and the supernatant was collected for instrumental analysis. LODs are provided in **Supplementary Table 2**.

### 2.4 Statistical analysis

The characteristics of the study population, distributions of seminal plasma metal concentrations, urinary oxidative stress (OS) biomarkers, and sperm quality parameters were summarized as mean ± standard deviation (SD) for continuous variables and as number (percentage) for categorical variables. The distributions of the seventeen seminal plasma metals were further described using geometric means (GM) and selected percentiles (P_5_, P_25_, P_50_, P_75_, and P_95_). Given the right-skewed distributions of metal concentrations, urinary OS biomarkers, and sperm quality parameters, natural logarithmic (ln) transformation was applied prior to statistical analyses. A multi-step statistical analysis was performed to minimize potential bias arising from reliance on a single analytical approach.

First, single-exposure models were constructed using multivariable linear regression to evaluate the associations between individual metal concentrations and sperm quality parameters. Second, multi-exposure models were constructed using weighted quantile sum (WQS) regression to assess the joint effects of metal mixtures on sperm quality parameters and to identify key contributors within the mixtures. Third, mediation analysis was conducted to examine the potential mediating role of urinary oxidative stress (OS) biomarkers in the associations between seminal plasma metal concentrations and sperm quality parameters. Detailed descriptions of these models are provided below.

Multivariable linear regression models were first used to evaluate the associations between seminal plasma metal concentrations (modeled as continuous variables), sperm quality parameters, and urinary oxidative stress (OS) biomarkers. Subsequently, participants were categorized into low, medium, and high exposure groups based on tertiles of metal concentrations, with the lowest tertile serving as the reference group. Tests for linear trend were then conducted by modeling the median value of each tertile as a continuous variable, with statistical significance assessed using the Wald test.

To further characterize the joint effects of exposure to seventeen seminal plasma metals on sperm quality, weighted quantile sum (WQS) regression was adopted as a flexible mixture model. In this framework, all seventeen metals were constrained to have effects in the same direction on sperm quality parameters, thereby reducing model dimensionality. Because WQS regression assumes directional homogeneity, analyses were conducted twice to evaluate associations in both directions: one model constrained the overall effect estimate (*β*_1_) to be positive, and the other constrained it to be negative. Metal concentrations were combined into a weighted index representing the overall effect of the metal mixture on sperm quality parameters. A total of 10,000 bootstrap samples were generated, with 60% of the data randomly assigned to the training set for weight estimation and the remaining 40% used for validation. Metals with final weights exceeding the predefined threshold were considered major contributors to the WQS index.

Lastly, the exploratory mediation analysis model was further conducted to examine whether oxidative stress (OS) indicators mediate the associations between metal exposure (independent variables) and sperm quality (dependent variables). This approach decomposes the total effect (TE) of exposure into direct and indirect components, thereby clarifying potential pathways linking exposure to sperm quality. The average direct effect (ADE) reflects the effect of exposure on sperm quality independent of the mediator, whereas the average causal mediation effect (ACME) quantifies the indirect effect of each metal on sperm quality through OS indicators. An ACME P-value < 0.05 was considered indicative of a statistically significant mediation effect.

All models were adjusted for potential confounders, including age, body mass index (BMI), education level, infertility type, alcohol consumption, and smoking status (20). Statistical analyses were conducted using R software (version 4.2.2) and SPSS (version 24.0). All tests were two-sided, and a P-value < 0.05 was considered statistically significant.

## 3 Results

### 3.1 Description of the population

The baseline characteristics and sperm quality parameters of the 425 male participants are provided in **Table 1**. The mean age (± *SD*) was 34.00 ± 6.01 years, and the mean BMI was 25.72 ± 3.66 kg/m². Regarding educational attainment, 62.62% of participants had a technical secondary school education or lower, whereas 20.24% held a bachelor’s degree or higher. More than half of the participants reported current smoking and alcohol consumption. Overall, 53.88% were classified as overweight or obese, and 46.82% had secondary infertility. The median values for sperm concentration (10^6^/mL), sperm DNA fragmentation index (%), sperm abnormality rate (%), sperm motility, and sperm progressive motility (%) were 66.06, 13.81, 95.07,64.90 and 62.22, respectively.

**Table 1:** Socio-demographic statistical analysis. (*N* = 425).

Characteristics	n[%]/mean(P25,P75)
Age	34.00± 6.01
Educational level	
Technical secondary school and below	263 (62.62%)
Junior college	72 (17.14%)
Bachelor’s degree or above	85 (20.24%)
Smoking status	
No	185 (43.53%)
< 1 days/week	13 (3.06%)
1–3 days/week	56 (13.18%)
> 3days/week	69 (16.24%)
Every day	102 (24%)
Alcohol consumption	
No	156 (36.71%)
< 1 time/week	205 (48.24%)
Every week	64 (15.06%)
BMI	25.72 ± 3.66
<18.5	12 (2.84%)
18.5–23.9	181 (42.89%)
≥24	229 (54.26%)
Infertility type	
Primary infertility	226 (53.18%)
Secondary infertility	199 (46.82%)
Sperm concentration (10^6^/mL)	66.06(32.20,93.02)
Sperm DNA fragmentation index (%)	13.81(5.88,18.00)
Sperm abnormality rate (%)	95.07(95.00,96.56)
Sperm total motility (%)	64.90(49.30,82.76)
Sperm progressive motility (%)	62.22(41.88,75.68)

### 3.2 Distribution of metals in seminal plasma

**Supplementary Table 3** shows the distribution of metal concentrations in seminal plasma. The geometric mean (*GM*) concentrations of Al, As, Ba, Cd, Co, Cr, Cu, Fe, Hg, Mn, Ni, Pb, Se, Sn, Sr, Tl, and V were 243.366, 2.019, 3.386, 0.616, 0.320, 5.382, 145.662, 476.053, 0.316, 10.420, 0.979, 1.245, 73.728, 2.953, 97.351, 0.174, and 1.226 μg/L, respectively. Detection rates were 100% for all metals except Sn (91.06%), Ni (99.29%), and Se (99.76%).

### 3.3 Associations between seminal plasma metals and sperm quality parameters

Linear regression analysis results for the association between metals exposure and sperm quality parameters are shown in **Supplementary Table 4**, with each metal modeled as both continuous and categorical variables. When metals were analyzed as continuous variables, Fe, As, Se, and Cd were positively associated with sperm concentration. In contrast, Mn, Fe, Co, and Cu were negatively correlated with both progressive and total motility. Finally, Sr was the only metal negatively correlated with sperm abnormality rate.

When metal concentrations in seminal plasma were categorized into tertiles (with the lowest tertile [T1] as the reference group), several significant associations with sperm quality parameters were observed. For sperm abnormality rate, both the medium (T2 vs T1: *β* = 0.037; 95% *CI*: 0.006 to 0.067) and highest tertiles (T3 vs T1: *β* = 0.045; 95% *CI*: 0.014 to 0.077) of Al were positively associated with sperm abnormality rate, with a significant dose–response trend across tertiles (*p* for trend = 0.005). For progressive motility, the highest tertile of Co (T3 vs T1: *β* = −0.338; 95% *CI*: −0.479 to −0.197) and Cu (T3 vs T1: *β* = −0.148; 95% CI: −0.294 to −0.001) showed significant inverse associations, with significant trends across tertiles (*p* for trend < 0.001 and p for trend = 0.001, respectively). The medium tertile of Mn (T2 vs T1: *β* = −0.236; 95% *CI*: −0.379 to −0.060), Ni (T2 vs T1: *β* = −0.204; 95% *CI*: −0.349 to −0.094), and Tl (T2 vs T1: *β* = −0.171; 95% *CI*: −0.314 to −0.028) was also inversely associated with progressive motility (all *p* for trend < 0.05). In contrast, Al concentrations were positively associated with progressive motility, with significant associations observed in both the medium (T2 vs T1: *β* = 0.214; 95% *CI*: 0.071 to 0.358) and highest tertiles (T3 vs T1: *β* = 0.197; 95% *CI*: 0.047 to 0.348), accompanied by a significant dose–response trend (*p* for trend = 0.010). Positive associations with sperm concentration were observed for As (T3 vs T1: *β* = 0.278; 95% *CI*: 0.072 to 0.485; *p* for trend = 0.009), Cu (T2 vs T1: *β* = 0.232; 95% *CI*: 0.025 to 0.440; *p* for trend = 0.029), Fe (T3 vs T1: *β* = 0.335; 95% CI: 0.135 to 0.536; *p* for trend = 0.001), and Se (T3 vs T1: *β* = 0.417; 95% *CI*: 0.213 to 0.621; *p* for trend<0.001). Total motility was inversely associated with Mn (T3 vs T1: *β* = −0.186; 95% *CI*: −0.323 to −0.049; *p* for trend = 0.008), Co (T3 vs T1: *β* = −0.367; 95% *CI*: −0.501 to −0.233; *p* for trend<0.001), Ni (T2 vs T1: *β* = −0.252; 95% *CI*: −0.389 to −0.115; *p* for trend < 0.001), and Tl (T2 vs T1: *β* = −0.177; 95% *CI*: −0.314 to −0.040; *p* for trend = 0.012). In contrast, Al concentrations were positively associated with total motility, with significant associations observed in the highest tertiles (T3 vs T1: *β* = 0.184; 95% *CI*: 0.046 to 0.321), accompanied by a significant dose–response trend (p for trend = 0.009).

### 3.4 Joint effects of seminal plasma metal mixtures on sperm quality: WQS analyses

**Figure 1:**
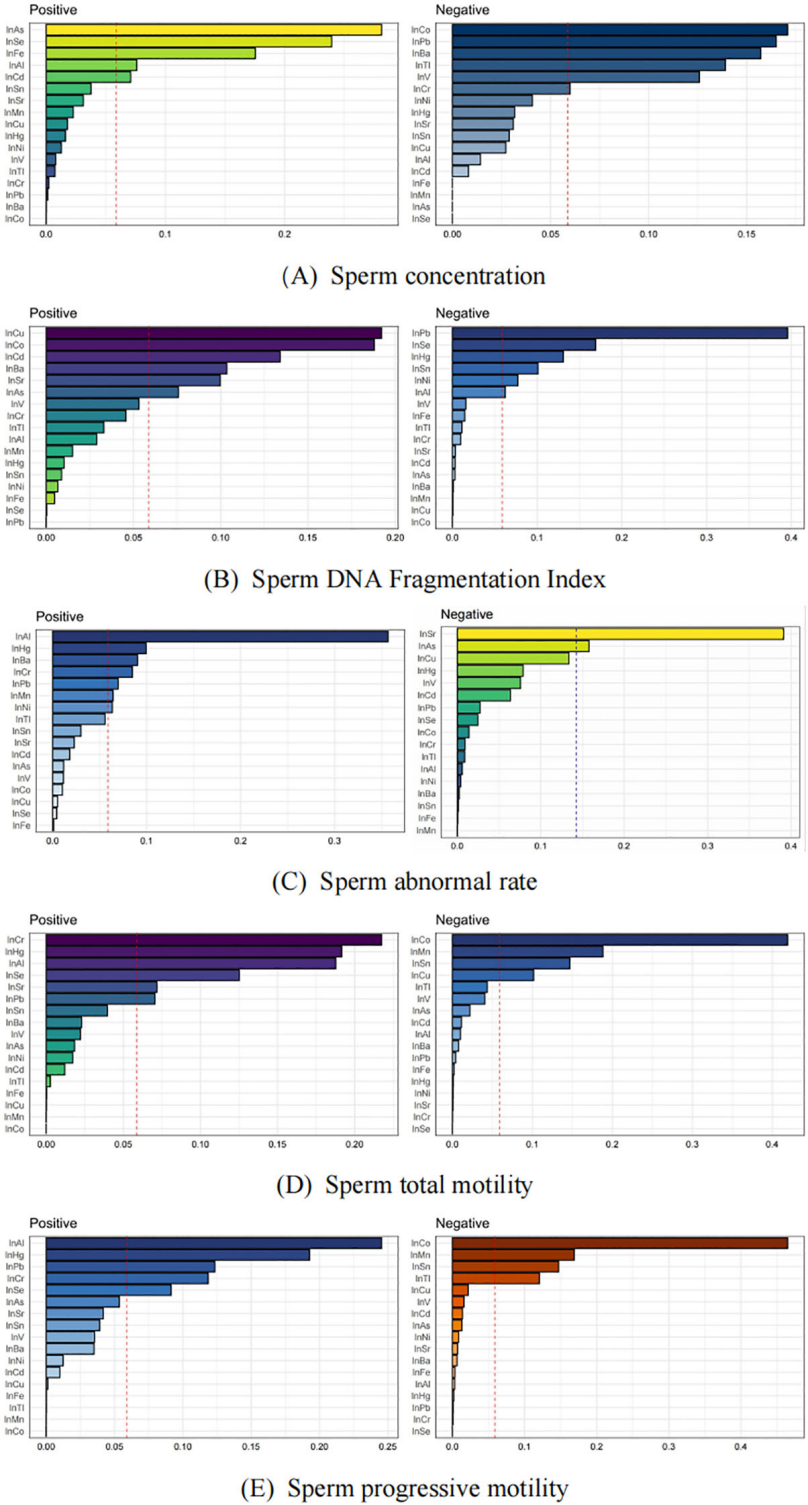
WQS models of the association between metal mixture exposure and sperm quality. Legend 1: Weighted Quantile Sum (WQS) regression analysis of the associations between metal mixtures and sperm quality parameters. **(A)** Sperm concentration; **(B)** Sperm DNA Fragmentation Index (DFI); **(C)** Abnormal sperm morphology; **(D)** Total sperm motility; and **(E)** Progressive sperm motility. For each sperm quality outcome, the left panel (Positive) shows the estimated weights of individual metals in the positive-direction WQS model, whereas the right panel (Negative) shows the estimated weights in the negative-direction WQS model. The y-axis lists the metal elements included in the mixture analysis, and the x-axis represents the relative contribution (weight) of each metal to the overall WQS index. The red vertical dashed line represents the equal-weight reference value (1/17 = 0.0588), calculated based on the 17 metals included in the WQS model.

In this study, seventeen seminal plasma metals were considered as a mixture, and their joint associations with sperm quality parameters were evaluated using WQS regression. In the positive-direction WQS model, a one-quantile increase in the WQS index was significantly associated with increased sperm abnormality rate (β = 0.014; 95% CI: 0.004 to 0.024; P = 0.007). The mixture effect was mainly driven by Al (weight = 0.357), followed by Hg (0.099) and Ba (0.090) (**Figure 1**). Regarding sperm motility, the metal mixture showed a significant negative association with total motility (β = −0.188; 95% CI: −0.309 to −0.067; P = 0.003). Notably, Co was identified as the primary contributor to this negative mixture effect (weight = 0.419). Among the positively weighted metals, Se contributed most to this association (weight = 0.229), whereas Co (weight = 0.321) was the main contributor in the negative direction. In line with these findings, Se also exhibited one of the highest positive weights in the positive-direction WQS model, while Co showed the highest negative weight in the negative-direction WQS model.

### 3.5 Associations between seminal plasma metal mixtures and urinary oxidative stress indicators

**Figure 2:**
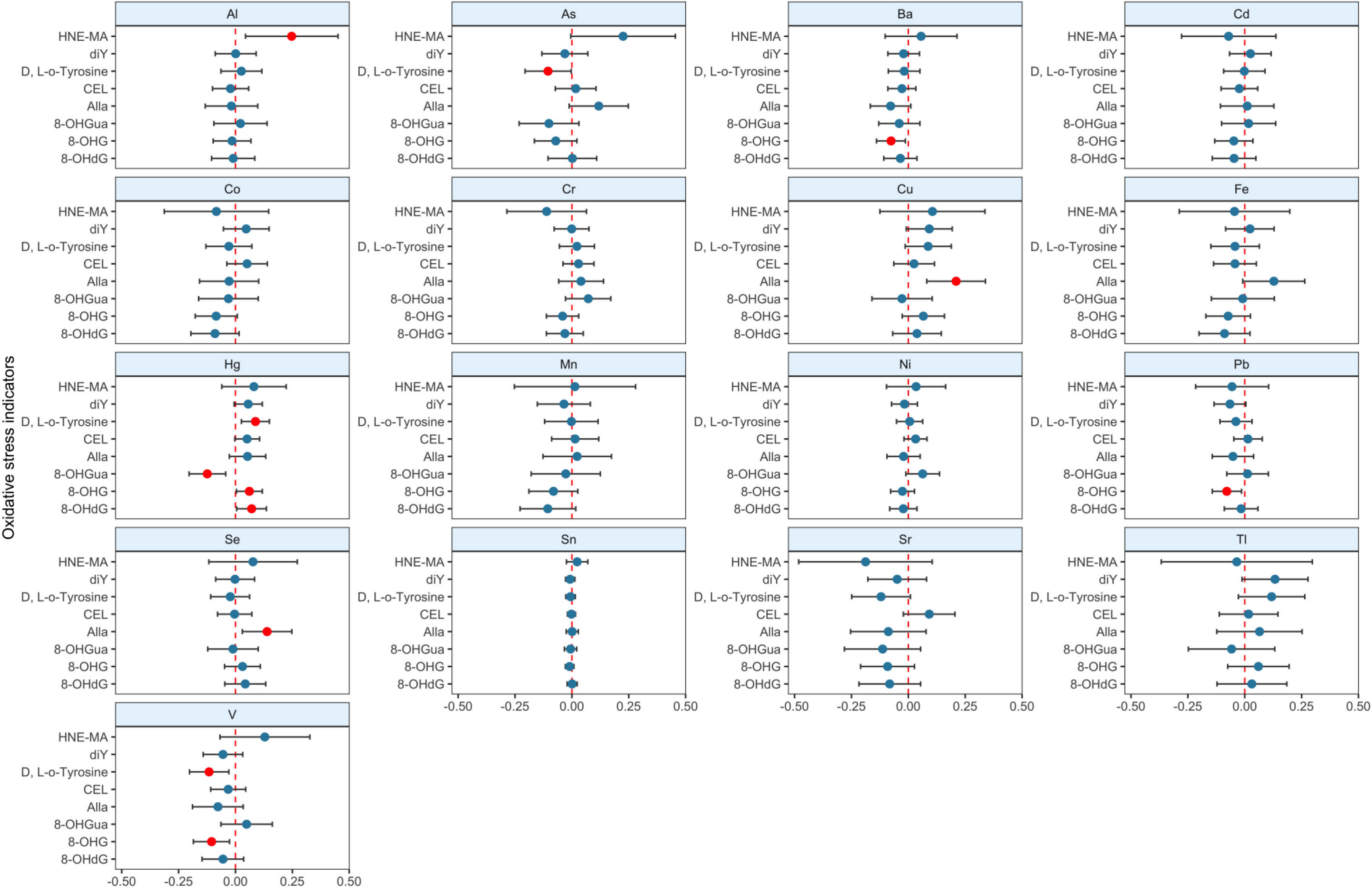
Associations between trace element concentrations and oxidative stress biomarkers. Legend 2: Regression coefficients (*β*) and 95% confidence intervals were derived from multivariable linear regression models, adjusted for potential confounding factors. Red dots indicate statistically significant associations (*P* < 0.05); blue dots indicate non-significant associations (*P* ≥ 0.05).

As shown in **Figure 2**, only a limited number of statistically significant associations were observed between seminal metal concentrations and oxidative stress biomarkers. Specifically, Al was positively associated with HNE-MA. Cu and Se were both positively associated with Alla. Hg showed significant positive associations with D,L-o-Tyrosine, 8-OHG, and 8-OHdG, but a negative association with 8-OHGua. In addition, As and V were negatively associated with D,L-o-Tyrosine, Ba and Pb were negatively associated with 8-OHG, and V was also negatively associated with 8-OHGua. No statistically significant associations were observed for the remaining metal–biomarker pairs.

### 3.6 Associations between urinary oxidative stress indicators and sperm quality parameters

**Table 2** presents the associations between oxidative stress markers and sperm quality parameters, CEL and Alla were both significantly negatively correlated with sperm abnormality rate (*β* = -0.028, 95% *CI*: -0.055 to 0.000, *p* = 0.048; *β* = -0.024, 95% *CI*: -0.042 to -0.006, *p* = 0.048, respectively), whereas diY was significantly positively correlated with sperm abnormality rate (*β* = 0.027, 95% *CI*: 0.004 to 0.051, *p* = 0.023). In addition, D,L-o-Tyrosine was significantly negatively associated with sperm concentration (*β* = -0.228, 95% *CI*: -0.383 to -0.073, *p* = 0.004). No significant associations were observed between the remaining oxidative stress markers and sperm quality parameters (p > 0.05).

**Table 2:** Associations between oxidative stress indicators and sperm quality parameters.

Semen quality parameters	Oxidative stress biomarkers	β (95% CI)	P
Sperm concentration	8-OHdG	-0.004 (-0.153,0.144)	0.953
	8-OHG	-0.061 (-0.23,0.108)	0.477
	8-OHGua	0.146 (0.026,0.266)*	**0.017**
	HNE-MA	0.002 (-0.067,0.071)	0.948
	diY	0.057 (-0.1,0.215)	0.475
	Alla	0.02 (-0.102,0.141)	0.753
	CEL	-0.039 (-0.217,0.139)	0.669
	D, L-o-Tyrosine	-0.228 (-0.383,-0.073)**	**0.004**
Sperm DNA Fragmentation Index	8-OHdG	-0.021 (-0.173,0.131)	0.786
	8-OHG	-0.111 (-0.285,0.063)	0.210
	8-OHGua	-0.143 (-0.267,-0.02)*	**0.023**
	HNE-MA	-0.03 (-0.101,0.04)	0.396
	diY	0.071 (-0.09,0.233)	0.383
	Alla	-0.072 (-0.195,0.05)	0.247
	CEL	0.06 (-0.122,0.242)	0.516
	D, L-o-Tyrosine	-0.077 (-0.235,0.081)	0.338
Sperm abnormality rate	8-OHdG	-0.009 (-0.031,0.014)	0.443
	8-OHG	-0.023 (-0.049,0.003)	0.086
	8-OHGua	-0.01 (-0.027,0.008)	0.296
	HNE-MA	-0.01 (-0.02,0)	0.057
	diY	0.027 (0.004,0.051)*	**0.023**
	Alla	-0.024 (-0.042,-0.006)*	**0.011**
	CEL	-0.028 (-0.055,0)*	**0.048**
	D, L-o-Tyrosine	-0.009 (-0.032,0.014)	0.440
Sperm progressive motility	8-OHdG	0.038 (-0.065,0.142)	0.467
	8-OHG	0.027 (-0.092,0.146)	0.654
	8-OHGua	0.05 (-0.034,0.135)	0.239
	HNE-MA	-0.034 (-0.082,0.014)	0.168
	diY	0.023 (-0.087,0.133)	0.681
	Alla	-0.075 (-0.161,0.01)	0.085
	CEL	0.02 (-0.104,0.144)	0.749
	D, L-o-Tyrosine	-0.046 (-0.156,0.064)	0.411
Sperm total motility	8-OHdG	0.064 (-0.035,0.163)	0.207
	8-OHG	0.052 (-0.062,0.166)	0.367
	8-OHGua	0.068 (-0.012,0.149)	0.097
	HNE-MA	-0.024 (-0.071,0.022)	0.298
	diY	0.015 (-0.09,0.121)	0.773
	Alla	-0.003 (-0.085,0.079)	0.935
	CEL	0.025 (-0.094,0.143)	0.679
	D, L-o-Tyrosine	-0.048 (-0.153,0.056)	0.364

8-OHdG, 8-hydroxy-2′-deoxyguanosine; 8-OHG, 8-hydroxyguanosine; 8-OHGua, 8-hydroxyguanine HNE-MA, 4-hydroxy-2-nonenal-mercapturic acid; diY, dityrosine; Alla, allantoin; CEL, N6-carboxyethyl-lysine; D, L-o-tyrosine: D, L-ortho-tyrosine, *P<0.05, **P<0.01. Bold values indicate statistically significant differences (P < 0.05).

### 3.7 Exploratory mediation analysis

**Figure 3:**
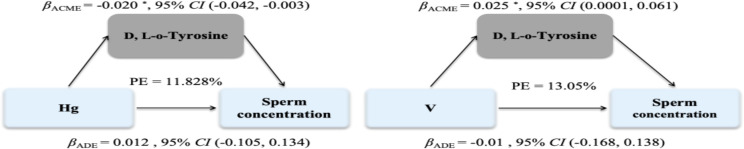
The exploratory mediating effects of oxidative stress markers in the association between metal elements and sperm concentration. Legend 3: *β*_ACME_, average causal mediation effect; *β*_ADE_, average direct effect; *PE*, proportion of total effect mediated, 95% *CI*, 95% confidence interval; *indicates statistically significant mediation effect (*P* < 0.05).

To preliminarily explore whether oxidative stress biomarkers may mediate the associations between seminal plasma metals and sperm quality, we performed exploratory mediation analyses. The results indicated that D,L-o-tyrosine significantly mediated the associations of seminal plasma Hg and V with sperm concentration, with mediated proportions of 11.8% and 13.0%, respectively. (**Figure 3**).

## 4 Discussion

This cohort study aimed to evaluate the associations between seminal plasma metal exposure and sperm quality and to further explore whether urinary oxidative stress biomarkers mediate these associations. First, we found that the levels of Co, Cu, and Mn in seminal plasma were negatively correlated with both sperm progressive motility and total motility. Additionally, a negative association was observed between seminal plasma Sr levels and the sperm abnormality rate. Finally, seminal plasma levels of Se and Fe were significantly and positively associated with sperm concentration.

In this study, seminal plasma Co and Mn levels were significantly and negatively associated with both total and progressive sperm motility. Mixture analysis further indicated that Co and Mn were major contributors to the observed mixture effects, as evidenced by their consistently higher weights in the WQS models. Several studies have provided evidence supporting our findings regarding Mn exposure and impaired male reproductive function. Epidemiological data indicate that occupational Mn exposure is associated with abnormal reproductive hormone profiles and reduced sperm motility (21). Experimental studies further demonstrate that Mn exposure induces oxidative damage, histopathological alterations, and endocrine disruption in the testes, ultimately impairing spermatogenesis and sperm quality in animal models (22). In addition, a review suggest that elevated Mn levels may suppress antioxidant enzyme activity, thereby enhancing oxidative stress, a key pathway implicated in sperm dysfunction (5).

Cobalt (Co) is an essential trace element primarily obtained through dietary intake and plays a physiological role as a component of vitamin B12 (23). In addition to its nutritional relevance, Co is extensively utilized in modern industrial applications, particularly in battery manufacturing and as a constituent of orthopedic implant materials (24, 25), thereby increasing the potential for occupational and environmental exposure. Previous study has reported that Co exposure in seminal plasma was negatively associated with sperm motility (26). This finding is consistent with our results, which showed that seminal plasma Co levels were negatively associated with both progressive and total sperm motility. Moreover, Co exhibited the highest weight in the WQS models, indicating that it was the major contributor to the mixed-metal exposure effects. In the reproductive context, this phenomenon may be attributed to the ability of Co to generate ROS, promote lipid peroxidation, and disrupt mitochondrial function, thereby exerting cytotoxic effects and compromising sperm integrity (27).

Regarding sperm concentration, our study observed positive associations between seminal plasma levels of As, Se, and Fe and sperm concentration. Consistently, a previous study reported that seminal plasma Se and Fe levels were positively associated with both sperm concentration and total sperm count (28). These associations may partly reflect the physiological roles of essential trace elements in male reproductive function. Se is an essential component of selenoproteins involved in antioxidant defense, redox regulation, sperm maturation, and spermatogenesis (29), whereas Fe participates in oxygen transport, mitochondrial respiration, energy metabolism, and cellular proliferation (30).

However, these positive associations should be interpreted cautiously. The biological effects of essential trace elements are concentration-dependent and may occur within a narrow physiological window. Excessive Fe may promote oxidative stress, redox imbalance, and lipid peroxidation (31) whereas excessive Se may also exert toxic effects and adversely affect reproductive function (32). Notably, in our study, the associations of Fe were not uniformly favorable across semen parameters: although Fe was positively associated with sperm concentration, it was inversely associated with total and progressive sperm motility in continuous models. Therefore, the observed positive associations of Fe and Se with sperm concentration should not be interpreted as evidence that higher levels are necessarily beneficial, nor as support for increased supplementation. Rather, they may reflect the complex and potentially biphasic effects of essential trace elements on different domains of semen quality. Further prospective and mechanistic studies are needed to clarify the optimal concentration ranges of Fe and Se for male reproductive health.

In contrast to our findings, well-established evidence indicates that As exposure can impair sperm quality (33, 34). Experimental studies have demonstrated that As exposure impairs male reproductive function primarily through oxidative stress (35). In animal models, genotoxic stress triggered by ROS has been shown to mediate As-induced suppression of germ cell proliferation and deterioration of sperm quality (36). Additionally, As exposure has been reported to reduce the expression of key spermatogenesis-related genes, including ZMYND10, ZMYND15, and their downstream targets such as Tnp1, thereby disrupting testicular function in male rats (37). However, in contrast to previous evidence suggesting adverse reproductive effects of As exposure, our results showed a positive statistical association between seminal plasma As levels and sperm concentration. This finding should not be interpreted as evidence of a beneficial effect of As, but rather as an observational association that requires cautious interpretation and further validation This discrepancy may be attributable to differences in exposure levels. At relatively low doses, As exposure may induce adaptive or compensatory responses in the organism, potentially resulting in a non-linear or hormetic effect (38). Therefore, the specific effects of metal exposure on sperm quality warrant further investigation in large-scale, multicenter studies incorporating mechanistic analyses.

Redox homeostasis is essential for normal male reproductive function. At physiological levels, reactive oxygen species (ROS) are not merely harmful by-products but act as important signaling molecules involved in spermatogenesis and fertilization-related processes (39). Low to moderate ROS levels contribute to germ cell proliferation and differentiation, spermatogonial stem cell self-renewal, sperm capacitation, hyperactivation, acrosome reaction, sperm–oocyte interaction, and zona pellucida binding (40, 41). These processes are partly mediated through ROS-dependent signaling pathways, including mitochondrial regulation, calcium influx, cyclic adenosine monophosphate/protein kinase A (cAMP/PKA) activation, tyrosine phosphorylation, and membrane remodeling (42, 43). However, when ROS levels exceed physiological requirements or when redox regulation is disrupted, oxidative stress may contribute to male reproductive dysfunction.

Excessive generation of reactive oxygen species (ROS) has been shown to compromise sperm quality by reducing total sperm count, motility, concentration, morphology, and viability, while increasing DNA fragmentation (44). These effects may be attributed to oxidative stress–induced damage, including lipid peroxidation of the sperm plasma membrane, increased fragmentation of both nuclear and mitochondrial DNA, and disruption of transcriptional homeostasis (45). Consistent with these findings, this study revealed that the oxidative stress biomarker diY was positively associated with sperm abnormality rate, while D,L-o-Tyrosine was significantly and negatively associated with sperm concentration. However, this study also found that urinary levels of CEL and Alla were significantly negatively correlated with sperm abnormality rate. The inconsistency in these results may be related to differences in sample type and collection time. Because oxidative stress has been proposed as a potential mechanism linking metal exposure to impaired sperm quality (45). We further evaluated whether urinary oxidative stress biomarkers could account for part of these associations. Although D,L-o-tyrosine showed potential indirect effects in the associations of seminal plasma Hg and V with sperm concentration, with estimated mediated proportions of 11.8% and 13.0%, respectively, this finding should be interpreted cautiously. Several factors may explain the limited mediation evidence. First, the exposure, mediator, and outcome were measured in different biological matrices: metals were assessed in seminal plasma, oxidative stress biomarkers were measured in urine, and sperm quality parameters were obtained from semen analysis. This matrix inconsistency may weaken the biological plausibility of a direct mediation pathway. Second, in the male reproductive system, ROS may arise from both systemic and local sources. External exposures, including smoking, alcohol consumption, environmental pollutants, and heavy metals, can promote systemic oxidative stress, whereas oxidative stress within the reproductive tract is more likely to exert a direct effect on fertility (47). In this context, urinary oxidative stress biomarkers should be interpreted with caution, as they may reflect systemic oxidative damage but may not adequately represent the local redox status of semen, testicular tissue, or the epididymal compartment (46). Therefore, the mediation analysis should be regarded as exploratory rather than confirmatory. The exploratory mediation evidence does not exclude a role of oxidative stress in metal-related male reproductive toxicity but suggests that future studies using seminal or reproductive tract-specific oxidative stress biomarkers are warranted to clarify whether oxidative stress links metal exposure to male reproductive system injury.

This study has several strengths. First, we assessed exposure levels of multiple metal biomarkers and analyzed their associations with sperm quality both individually and in combination, providing a more comprehensive perspective than previous research. Second, by using seminal plasma as the matrix for measuring metal concentrations, our study more accurately reflects the internal exposure level within the male reproductive system, thereby enhancing the biological plausibility and persuasiveness of our findings. Nevertheless, there are certain limitations that need to be considered. First, while multiple previous studies have suggested that oxidative stress markers may mediate the effects of metals on sperm quality, this phenomenon was not observed in our findings. This lack of observation could be attributed to issues related to sample collection and measurement, or to the fact that the oxidative stress markers were measured in urine rather than seminal plasma, which may not fully capture the oxidative stress status within the male reproductive system. In addition, 154 participants were excluded from the oxidative stress biomarker analysis because urine samples were not available; therefore, potential selection bias related to incomplete biospecimen collection cannot be completely ruled out.

## Data Availability

The raw data supporting the conclusions of this article will be made available by the authors, without undue reservation.
