# Supplementary material for: Associations of seminal plasma metal mixtures with sperm quality parameters and exploratory mediation analysis of urinary oxidative stress biomarkers: a cross-sectional study from the RHCC-AC preconception cohort
Source: Front Endocrinol (Lausanne). 2026 Jul 8;17:1861843. doi: 10.3389/fendo.2026.1861843 (PMC13388122; doi:10.3389/fendo.2026.1861843)
Supplement: Supplementary file 1 [file Table1.docx]

**Supplementary Materials Table of Contents**

Supplementary Table 1 Metal elements of detection limits, linear ranges, accuracy and precision.

Supplementary Table 2 Oxidative stress markers of detection limits, linear ranges, accuracy and precision.

Supplementary Table 3 Distribution of metals in seminal plasma.

Supplementary Table 4 Associations between seminal plasma metals and sperm quality parameters.

**Supplementary Table 1** Metal elements of detection limits, linear ranges, accuracy and precision.

| Elements | LOD (ng/mL) | Linearity range (ng/mL) | Adding standard recover (%) | Within-day precisio（%） | Inter-day precision（%） |
| --- | --- | --- | --- | --- | --- |
| Fe | 0.0100 | 2～15 | 102 | 21 | 29 |
| Cu | 0.0018 | 2～15 | 98 | 5 | 19 |
| Zn | 0.0180 | 5～25 | 96 | 3 | 6 |
| Se | 0.0240 | 0.5～5 | 92 | 6 | 7 |
| Co | 0.0002 | 0.5～5 | 104 | 3 | 7 |
| Mo | 0.0006 | 2～15 | 113 | 3 | 6 |
| V | 0.0003 | 0.5～5 | 109 | 0 | 7 |
| Ni | 0.0012 | 0.5～5 | 102 | 3 | 9 |
| Mn | 0.0017 | 0.5～5 | 101 | 9 | 13 |
| Cr | 0.0020 | 0.5～5 | 105 | 6 | 14 |
| As | 0.0009 | 0.5～5 | 96 | 3 | 8 |
| Cd | 0.0003 | 0.5～5 | 96 | 7 | 13 |
| Pb | 0.0021 | 0.5～5 | 105 | 5 | 15 |
| Tl | 0.0001 | 0.5～5 | 102 | 8 | 7 |
| Sn | 0.0034 | 0.5～5 | 108 | 8 | 20 |
| Ba | 0.0053 | 0.5～5 | 109 | 4 | 10 |
| Cs | 0.0001 | 0.5～5 | 107 | 2 | 4 |
| Sb | 0.0075 | 0.5～5 | 120 | 1 | 17 |
| Sr | 0.0004 | 2～15 | 104 | 1 | 4 |

Note: LOQ(Limit of Detection).

**Supplementary Table 2** Oxidative stress markers of detection limits, linear ranges, accuracy and precision.

| Oxidative stress markers | LOQ(ng/mL) | Linearity rang(ng/mL) | Adding standard recovery(%) | Within-day precision(%) | Inter-day precision(%) |
| --- | --- | --- | --- | --- | --- |
| 8-OHdG | 0.05 | 0.05～10 | 71.20 | 2.59 | 3.83 |
| 8-OHG | 0.02 | 0.01～10 | 82.28 | 2.59 | 1.67 |
| 4-HNEMA | 0.20 | 0.2～500 | 71.11 | 2.05 | 1.83 |
| Alla | 22.0 | 1.1～11000 | 91.08 | 1.29 | 3.19 |
| CEL | 2.00 | 1～500 | 110.50 | 0.74 | 4.14 |
| diY | 0.44* | 0.2～20 | 117.95 | 1.78 | 4.66 |
| 8-OHGua | 10.6* | 0.5～200 | 95.39 | 3.82 | 4.13 |
| D,L-o-Tyrosine | 0.77* | 0.1～20 | 137.81 | 1.62 | 1.24 |

Note: LOQ(Limit of Detection), *: LOQ = sample blank value + 3 × standard deviation of the sample blank.

**Supplementary Table 3** Distribution of metals in seminal plasma.

| Elements | Detection rates (% ) | Geometric Mean | P5 | P25 | P50 | P75 | P95 |
| --- | --- | --- | --- | --- | --- | --- | --- |
| Al | 100 | 243.366 | 114.041 | 161.457 | 197.866 | 356.371 | 748.414 |
| As | 100 | 2.019 | 0.878 | 1.411 | 1.993 | 2.740 | 5.073 |
| Ba | 100 | 3.386 | 1.398 | 2.109 | 3.215 | 4.999 | 11.394 |
| Cd | 100 | 0.616 | 0.185 | 0.397 | 0.622 | 1.001 | 1.800 |
| Co | 100 | 0.320 | 0.152 | 0.240 | 0.312 | 0.403 | 0.666 |
| Cr | 100 | 5.382 | 2.219 | 3.464 | 4.854 | 7.448 | 16.488 |
| Cu | 100 | 145.662 | 73.541 | 100.409 | 137.875 | 188.514 | 366.346 |
| Fe | 100 | 476.053 | 243.790 | 356.276 | 450.370 | 619.073 | 993.004 |
| Hg | 100 | 0.316 | 0.105 | 0.180 | 0.266 | 0.454 | 1.871 |
| Mn | 100 | 10.420 | 5.394 | 7.866 | 10.352 | 13.642 | 20.575 |
| Ni | 99.29 | 0.979 | 0.310 | 0.662 | 0.954 | 1.604 | 3.890 |
| Pb | 100 | 1.245 | 0.470 | 0.765 | 1.077 | 1.791 | 4.135 |
| Se | 99.76 | 73.728 | 24.917 | 53.731 | 80.712 | 110.144 | 168.731 |
| Sn | 91.06 | 2.953 | 0.002 | 2.965 | 7.049 | 12.212 | 20.739 |
| Sr | 100 | 97.351 | 51.213 | 72.836 | 94.260 | 130.111 | 191.777 |
| Tl | 100 | 0.174 | 0.099 | 0.133 | 0.172 | 0.220 | 0.316 |
| V | 100 | 1.226 | 0.495 | 0.827 | 1.189 | 1.585 | 3.962 |

**Supplementary Table 4** Associations between seminal plasma metals and sperm quality parameters.

| Sperm  quality parameters | Metals | Continuous variables | Tertiles | | *P*  for trend |
| --- | --- | --- | --- | --- | --- |
|  |  |  | T2 vs T1 | T3 vs T1 |  |
| Sperm concentration | Al | 0.121 (-0.028,0.269） | -0.012 (-0.218, 0.194) | 0.079 (-0.139, 0.297) | 0.482 |
|  | As | 0.22 (0.059,0.382）** | 0.042 (-0.162, 0.246) | 0.278 (0.072, 0.485)** | 0.009 |
|  | Ba | -0.028 (-0.141,0.085） | -0.162 (-0.366, 0.041) | -0.037 (-0.243, 0.169) | 0.721 |
|  | Cd | 0.159 (0.013,0.305）* | 0.154 (-0.055, 0.363) | 0.230 (-0.004, 0.463) | 0.052 |
|  | Co | -0.082 (-0.244,0.08） | 0.017 (-0.189, 0.223) | -0.049 (-0.255, 0.157) | 0.637 |
|  | Cr | -0.035 (-0.16,0.089） | 0.004 (-0.202, 0.210) | -0.022 (-0.227, 0.183) | 0.836 |
|  | Cu | 0.087 (-0.077,0.251） | 0.232 (0.025, 0.440)* | 0.158 (-0.050, 0.367) | 0.149 |
|  | Fe | 0.188 (0.016,0.36）* | 0.026 (-0.178, 0.229) | 0.335 (0.135, 0.536)** | 0.001 |
|  | Hg | -0.006 (-0.108,0.095） | 0.130 (-0.074, 0.334) | 0.112 (-0.096, 0.320) | 0.28 |
|  | Mn | 0.083 (-0.106,0.271） | -0.093 (-0.300, 0.114) | 0.064 (-0.141, 0.269) | 0.524 |
|  | Ni | 0.063 (-0.029,0.155） | -0.157 (-0.363, 0.050) | 0.020 (-0.185, 0.225) | 0.864 |
|  | Pb | -0.063 (-0.177,0.052） | 0.063 (-0.138, 0.265) | -0.065 (-0.269, 0.139) | 0.535 |
|  | Se | 0.256 (0.121,0.391）** | 0.197 (-0.006, 0.400) | 0.417 (0.213, 0.621)** | <0.001 |
|  | Sn | -0.005 (-0.039,0.029） | 0.150 (-0.061, 0.362) | 0.070 (-0.144, 0.283) | 0.546 |
|  | Sr | 0.074 (-0.134,0.281） | 0.068 (-0.138, 0.274) | 0.037 (-0.168, 0.243) | 0.714 |
|  | Tl | -0.026 (-0.262,0.21） | -0.054 (-0.259, 0.150) | 0.064 (-0.142, 0.270) | 0.541 |
|  | V | 0.017 (-0.125,0.159） | 0.017 (-0.189, 0.223) | -0.028 (-0.240, 0.184) | 0.793 |
| Sperm DNA fragmentation Index | Al | -0.094 (-0.243,0.055） | -0.129 (-0.340, 0.081) | -0.268 (-0.489, -0.046)* | 0.018 |
|  | As | 0.151 (-0.015,0.317） | -0.064 (-0.272, 0.145) | 0.098 (-0.113, 0.308) | 0.359 |
|  | Ba | 0.037 (-0.077,0.15） | 0.164 (-0.043, 0.370) | 0.087 (-0.124, 0.297) | 0.418 |
|  | Cd | 0.167 (0.019,0.316）* | 0.060 (-0.155, 0.275) | 0.209 (-0.027, 0.445) | 0.085 |
|  | Co | 0.114 (-0.048,0.276） | 0.012 (-0.196, 0.219) | 0.192 (-0.017, 0.401) | 0.074 |
|  | Cr | 0.049 (-0.077,0.176） | 0.058 (-0.152, 0.268) | 0.110 (-0.096, 0.317) | 0.295 |
|  | Cu | 0.211 (0.044,0.377）* | 0.043 (-0.164, 0.249) | 0.186 (-0.026, 0.399) | 0.088 |
|  | Fe | 0.059 (-0.118,0.237） | 0.085 (-0.125, 0.294) | 0.107 (-0.101, 0.315) | 0.317 |
|  | Hg | -0.005 (-0.11,0.1） | 0.035 (-0.171, 0.240) | -0.068 (-0.278, 0.143) | 0.553 |
|  | Mn | 0.257 (0.067,0.448）** | 0.142 (-0.066, 0.349) | 0.289 (0.084, 0.495)** | 0.006 |
|  | Ni | -0.027 (-0.12,0.066） | 0.133 (-0.077, 0.344) | 0.145 (-0.064, 0.354) | 0.172 |
|  | Pb | -0.122 (-0.235,-0.009） | 0.020 (-0.181, 0.222) | -0.236 (-0.443, -0.028)* | 0.028 |
|  | Se | -0.045 (-0.184,0.094） | -0.114 (-0.322, 0.094) | -0.048 (-0.259, 0.163) | 0.654 |
|  | Sn | -0.027 (-0.062,0.008） | -0.151 (-0.366, 0.065) | -0.092 (-0.309, 0.124) | 0.42 |
|  | Sr | 0.361 (0.153,0.569）** | 0.189 (-0.020, 0.397) | 0.287 (0.082, 0.491)** | 0.006 |
|  | Tl | 0.203 (-0.036,0.442） | 0.060 (-0.147, 0.267) | 0.198 (-0.012, 0.408) | 0.065 |
|  | V | 0.002 (-0.141,0.145） | 0.060 (-0.150, 0.270) | -0.067 (-0.281, 0.146) | 0.532 |
| Sperm abnormal rate | Al | 0.019 (-0.002,0.039） | 0.037 (0.006, 0.067)* | 0.045 (0.014, 0.077)** | 0.005 |
|  | As | 0.004 (-0.02,0.028） | 0.007 (-0.024, 0.037) | -0.011 (-0.042, 0.019) | 0.449 |
|  | Ba | 0.011 (-0.006,0.028） | -0.015 (-0.044, 0.015) | 0.019 (-0.012, 0.049) | 0.251 |
|  | Cd | 0.003 (-0.019,0.024） | -0.010 (-0.041, 0.022) | 0.010 (-0.024, 0.044) | 0.561 |
|  | Co | -0.01 (-0.033,0.014） | -0.007 (-0.037, 0.023) | -0.011 (-0.041, 0.019) | 0.473 |
|  | Cr | 0.005 (-0.013,0.023） | 0.026 (-0.004, 0.056) | 0.023 (-0.007, 0.053) | 0.129 |
|  | Cu | 0.002 (-0.021,0.026） | -0.013 (-0.043, 0.018) | -0.005 (-0.036, 0.025) | 0.729 |
|  | Fe | -0.001 (-0.026,0.024） | 0.003 (-0.027, 0.033) | -0.013 (-0.043, 0.017) | 0.385 |
|  | Hg | 0.013 (-0.002,0.029） | 0.028 (-0.001, 0.058) | 0.022 (-0.009, 0.052) | 0.155 |
|  | Mn | -0.009 (-0.037,0.018） | 0.001 (-0.029, 0.032) | -0.010 (-0.040, 0.020) | 0.522 |
|  | Ni | -0.003 (-0.016,0.01） | 0.007 (-0.023, 0.038) | -0.008 (-0.038, 0.023) | 0.624 |
|  | Pb | 0.007 (-0.009,0.023） | -0.008 (-0.038, 0.021) | 0.011 (-0.019, 0.041) | 0.471 |
|  | Se | 0.001 (-0.019,0.02） | 0.013 (-0.017, 0.043) | -0.013 (-0.043, 0.018) | 0.397 |
|  | Sn | 0.001 (-0.004,0.006） | 0.003 (-0.029, 0.034) | -0.009 (-0.040, 0.022) | 0.563 |
|  | Sr | -0.031 (-0.06,-0.001）* | -0.004 (-0.035, 0.026) | -0.016 (-0.046, 0.014) | 0.288 |
|  | Tl | 0.01 (-0.025,0.045） | -0.023 (-0.053, 0.006) | 0.006 (-0.024, 0.037) | 0.667 |
|  | V | 0.012 (-0.008,0.032） | -0.005 (-0.035, 0.026) | 0.009 (-0.022, 0.040) | 0.551 |
| Sperm total motility | Al | 0.005 (-0.095,0.105） | 0.184 (0.046, 0.321)** | 0.145 (-0.000, 0.289) | 0.046 |
|  | As | -0.047 (-0.157,0.062） | 0.085 (-0.054, 0.223) | 0.026 (-0.114, 0.166) | 0.718 |
|  | Ba | -0.002 (-0.082,0.079） | -0.080 (-0.216, 0.056) | -0.041 (-0.181, 0.099) | 0.549 |
|  | Cd | -0.016 (-0.114,0.083） | -0.028 (-0.171, 0.114) | -0.004 (-0.162, 0.153) | 0.948 |
|  | Co | -0.21 (-0.32,-0.1） | -0.107 (-0.240, 0.026) | -0.367 (-0.501, -0.233)** | <0.001 |
|  | Cr | 0.015 (-0.068,0.098） | -0.007 (-0.145, 0.131) | -0.029 (-0.167, 0.109) | 0.679 |
|  | Cu | -0.116 (-0.226,-0.007） | -0.026 (-0.165, 0.114) | -0.132 (-0.272, 0.009) | 0.064 |
|  | Fe | -0.134 (-0.249,-0.019） | -0.072 (-0.211, 0.067) | -0.072 (-0.208, 0.065) | 0.308 |
|  | Hg | 0.037 (-0.031,0.105） | -0.028 (-0.166, 0.110) | 0.068 (-0.070, 0.207) | 0.35 |
|  | Mn | -0.186 (-0.311,-0.06） | -0.064 (-0.202, 0.074) | -0.186 (-0.323, -0.049)** | 0.008 |
|  | Ni | 0.014 (-0.048,0.076） | -0.252 (-0.389, -0.115)** | -0.060 (-0.196, 0.075) | 0.371 |
|  | Pb | 0.013 (-0.073,0.099） | -0.020 (-0.156, 0.115) | -0.007 (-0.145, 0.131) | 0.917 |
|  | Se | 0 (-0.093,0.092） | 0.107 (-0.032, 0.246) | 0.009 (-0.131, 0.149) | 0.936 |
|  | Sn | 0.004 (-0.019,0.026） | 0.057 (-0.085, 0.198) | -0.083 (-0.225, 0.060) | 0.244 |
|  | Sr | -0.122 (-0.26,0.017） | 0.011 (-0.126, 0.149) | -0.096 (-0.235, 0.042) | 0.182 |
|  | Tl | -0.088 (-0.246,0.07） | -0.177 (-0.314, -0.040)* | -0.081 (-0.219, 0.056) | 0.255 |
|  | V | -0.004 (-0.1,0.091） | 0.045 (-0.093, 0.182) | 0.032 (-0.111, 0.174) | 0.664 |
| Sperm progressive motility | Al | 0.022 (-0.082,0.127） | 0.214 (0.071, 0.358)** | 0.197 (0.047, 0.348)* | 0.010 |
|  | As | -0.057 (-0.171,0.056） | 0.099 (-0.045, 0.243) | -0.023 (-0.169, 0.123) | 0.759 |
|  | Ba | 0.017 (-0.067,0.102） | -0.017 (-0.159, 0.126) | -0.000 (-0.146, 0.146) | 0.994 |
|  | Cd | -0.019 (-0.122,0.084） | -0.031 (-0.180, 0.118) | -0.040 (-0.205, 0.124) | 0.627 |
|  | Co | -0.185 (-0.301,-0.069）** | -0.067 (-0.207, 0.073) | -0.338 (-0.479, -0.197)** | <0.001 |
|  | Cr | 0.013 (-0.074,0.1） | -0.017 (-0.161, 0.128) | -0.054 (-0.198, 0.090) | 0.465 |
|  | Cu | -0.126 (-0.24,-0.011）* | -0.082 (-0.227, 0.064) | -0.148 (-0.294, -0.001) | 0.049 |
|  | Fe | -0.142 (-0.262,-0.021）* | -0.055 (-0.200, 0.090) | -0.105 (-0.247, 0.038) | 0.151 |
|  | Hg | 0.043 (-0.028,0.114） | -0.031 (-0.175, 0.113) | 0.044 (-0.101, 0.189) | 0.573 |
|  | Mn | -0.216 (-0.347,-0.085）** | -0.065 (-0.208, 0.079) | -0.236 (-0.379, -0.094)** | 0.001 |
|  | Ni | 0.015 (-0.05,0.08） | -0.204 (-0.349, -0.060)** | -0.029 (-0.172, 0.113) | 0.671 |
|  | Pb | 0.011 (-0.079,0.1） | -0.033 (-0.174, 0.109) | -0.037 (-0.181, 0.107) | 0.615 |
|  | Se | -0.007 (-0.103,0.089） | 0.127 (-0.017, 0.272) | -0.028 (-0.174, 0.118) | 0.668 |
|  | Sn | 0.011 (-0.013,0.035） | 0.032 (-0.116, 0.180) | -0.085 (-0.234, 0.064) | 0.254 |
|  | Sr | -0.141 (-0.286,0.004） | -0.032 (-0.176, 0.112) | -0.104 (-0.250, 0.041) | 0.161 |
|  | Tl | -0.122 (-0.286,0.043） | -0.171 (-0.314, -0.028)* | -0.115 (-0.258, 0.029) | 0.12 |
|  | V | 0.015 (-0.084,0.115） | 0.110 (-0.034, 0.253) | 0.076 (-0.072, 0.225) | 0.314 |

Note: In the Linear regression analysis, trace metal elements were modeled as both continuous and categorical variables(tertiles).

*P<0.05, **P<0.01
